# Monitoring Antimicrobial Use and Resistance: Comparison with a National Benchmark on Reducing Vancomycin Use and Vancomycin-Resistant Enterococci

**DOI:** 10.3201/eid0807.010465

**Published:** 2002-07

**Authors:** Scott K. Fridkin, Rachel Lawton, Jonathan R. Edwards, Fred C. Tenover, John E. McGowan, Robert P. Gaynes, the Intensive Care Antimicrobial Resistance Epidemiology (ICARE) Project, and the National Nosocomial Infections Surveillance (NNIS) System Hospitals

**Affiliations:** * Centers for Disease Control and Prevention, Atlanta, Georgia, USA; † Emory University, Atlanta, Georgia, USA

**Keywords:** antibiotic resistance, nosocomial infections, surveillance, benchmarking, quality

## Abstract

To determine if local monitoring data on vancomycin use directed quality improvement and decreased vancomycin use or vancomycin-resistant enterococci (VRE), we analyzed data from 50 intensive-care units (ICUs) at 20 U.S. hospitals reporting data on antimicrobial-resistant organisms and antimicrobial agent use. We compared local data with national benchmark data (aggregated from all study hospitals). After data were adjusted for changes in prevalence of methicillin-resistant Staphylococcus aureus, changes in specific prescriber practice at ICUs were associated with significant decreases in vancomycin use (mean decrease -48 defined daily doses per 1,000 patient days, p<0.001). These ICUs also reported significant decreases in VRE prevalence compared with those not using unit-specific changes in practice (mean decrease of 7.5% compared with mean increase of 5.7%, p<0.001). In this study, practice changes focused towards specific ICUs were associated with decreases in ICU vancomycin use and VRE prevalence.

The emerging problem of antimicrobial resistance in bacterial pathogens is very complex (1,2). However, one common theme is that antecedent antimicrobial exposure exerts selective pressure favoring the emergence of resistance (2). Appropriate antimicrobial use is an integral component of any program to slow the emergence and spread of antimicrobial-resistant microorganisms in the health-care setting (1,3). The optimal methods to reduce inappropriate or excessive antimicrobial use will differ by institution. Although many possible interventions have been proposed (4), deciding which one is the most effective in a particular setting can be difficult. Despite guidelines from governmental and professional groups (3,5–7), many hospitals have yet to institute any antimicrobial use policies or programs to improve antimicrobial agent prescribing (8).

The Infectious Disease Society of America and the Society for Healthcare Epidemiology of America Joint Committee on the Prevention of Antimicrobial Resistance recently published guidelines for the prevention of antimicrobial resistance in hospitals (3). Two of the six broad recommendations were to establish a system for monitoring bacterial resistance and antibiotic use and to establish practice guidelines and other institutional policies to control the use of antibiotics and respond to data from the monitoring system. Responding to data from a local monitoring system, especially in the context of an external benchmark, has been a successful way to create practice changes to improve the quality of patient care (9,10). Efforts have been made to improve outcomes for hospitalized patients; success has been demonstrated with surgical site infections and more recently, with catheter-related bloodstream infections (11,12). In both examples, local infection rates are compared with those of a sample that serves as an external benchmark. Valid benchmarks for comparing antimicrobial use have not been well established (13). One example of hospitals establishing a monitoring and benchmarking system is Project Intensive Care Antimicrobial Resistance Epidemiology (ICARE), a collaborative study between the Hospital Infections Program (now the Division of Healthcare Quality Promotion) at the Centers for Disease Control and Prevention (CDC) and the Rollins School of Public Health of Emory University. During this 4-year study, a subset of hospitals participating in the National Nosocomial Infections Surveillance (NNIS) system monitored antimicrobial use. We present data from Project ICARE that demonstrate how hospitals used local data and national benchmark data to effect practice changes resulting in reduced vancomycin use and prevalence of vancomycin-resistant enterococci (VRE) in intensive-care units (ICUs).

## Methods

### Setting

Hospitals that participate in the ICU surveillance component of the NNIS system were invited to participate in the second (January 1996 through December 1997) and third (April 1998 through July 1999) phases of Project ICARE; 55 ICUs from 21 hospitals reported the required data to both the second and third phase of Project ICARE. The surveillance methods and definitions of the NNIS system (14,15) and Project ICARE (13) have been previously described. As participants in the NNIS system, ICUs had been previously categorized by the types of patients served: coronary (CCU), medical (MICU), general surgical (SICU), cardiothoracic, combined medical-surgical (<80% of patients can be classified into a single ICU patient type), neurosurgical, respiratory, trauma, burn, or other.

### Data Collection

Participating hospitals reported the grams of select antimicrobial agents administered to patients and the antimicrobial susceptibility results of isolates recovered from clinical specimens from hospitalized patients each month. Microbiologic data were aggregated for each ICU separately, all non-ICU inpatient wards combined, and all outpatient areas combined (e.g., units that perform same-day surgery, simple diagnostic procedures or therapy, urgent care, or emergency care). Pharmacy data were reported for the same hospital strata, except for outpatient areas for which pharmacy data were not available. Amounts of antimicrobial drugs reported were standardized by conversion to defined daily doses; for parenteral vancomycin, one daily dose was defined as 2 g. This analysis includes both parenteral and oral (2% of total vancomycin use) vancomycin.

The microbiology laboratory reported antimicrobial susceptibility results for all enterococci and *Staphylococcus aureus* isolates recovered from all clinical specimens, whether associated with hospital- or community-acquired infection or colonization. Duplicate isolates were excluded: these were defined as isolates of the same organism with the same antimicrobial resistance pattern recovered from the same patient, regardless of the site of isolation (e.g., blood, sputum, urine, or wound), during the same calendar month. Susceptibility reports from isolates obtained as part of infection-control surveillance were excluded. When excluding these surveillance isolates, VRE or methicillin-resistant *S. aureus* (MRSA) prevalence more closely reflects data routinely aggregated as part of the cumulative susceptibility report (i.e., cumulative antibiogram). The validity of the susceptibility data submitted by participating hospitals for VRE and MRSA has previously been confirmed through a proficiency testing program at these laboratories and by confirmatory testing at the ICARE reference laboratory of up to 20 VRE and 20 MRSA isolates from these hospitals (13,16). Enterococci were considered vancomycin resistant if the MIC of vancomycin was 32 µg/mL or if the zone diameter by disk diffusion was 14 mm. *S. aureus* were considered oxacillin (methicillin) resistant if the MIC of oxacillin was 4 µg/mL or if the zone diameter by disk diffusion was 10 mm (17).

### Feedback Data

In October 1997, a report of local monitoring data for each hospital area compared to the national benchmark (i.e., aggregate summary data from all 41 Phase 2 ICARE hospitals, including 113 ICUs) was disseminated to each participating hospital (18). The aggregate benchmark data included numeric presentation of pooled means, medians, and key percentile distributions of the prevalence of selected antimicrobial-resistant organisms, stratified by ICU areas combined, non-ICU–inpatient areas combined, and outpatient areas combined (18). In addition, the data for antimicrobial agent use were stratified by the specific type of ICU (e.g., general-surgical separate from cardiothoracic ICU, non-ICU–inpatient areas combined, and outpatient areas combined) (18). Each individual hospital’s report included raw data and pooled means of the same target rates for each hospital area. Stratification of use and resistance prevalence by different hospital areas, as described, provided a valid benchmark by which hospitals were able to determine how their usage and resistance prevalence compared with the aggregate, when the data were adjusted for different patient populations in these different hospital areas.

To ascertain how the hospital infection-control staff used the feedback report, they were surveyed in September 1999. Information was collected on the use of the feedback report, recognition of problem pathogens or excessive use of specific antimicrobial agents, and specific practice changes. Questions were open-ended to include any change in infection-control practice (including hygienic practices, barrier precautions, and antimicrobial control practices) from baseline practice (i.e., during pre-intervention period), rather than a description of specific practices already in use. The infection-control practitioner overseeing the surveillance activities responded to the survey, with input from the infection-control committee, based on recollection or meeting minutes.

### Data Analysis

For this analysis, monthly data from each ICU were pooled for the entire study period and for each period of the study (i.e., pre-intervention and postintervention) by each ICU (data from non-ICU–inpatient areas and outpatient areas are not shown because of low statistical power). Pooled rates were calculated for prevalence of VRE (percentage), MRSA (percentage), and vancomycin use (defined daily doses/1,000 patient-days). For example, the pooled mean rate of vancomycin use was calculated for each ICU by dividing the total number of defined daily doses by the total number of patient-days reported over the study period by that ICU, multiplied by 1,000, and thus expressed as defined daily doses per 1,000 patient-days. If <10 *S. aureus* or enterococci isolates were tested for antimicrobial susceptibility from a specific ICU during the study period, that ICU was excluded from further analysis.

Data were analyzed by SAS Release 6.12 Software (SAS Institute Inc., Cary, NC). To assess the change in ICU-specific prevalence of MRSA, VRE, and vancomycin use, the pre-intervention rate was subtracted from the postintervention rate (i.e., difference in rates). Differences in the percent VRE and vancomycin use rate were evaluated by the paired t-test and further compared by type of practice change by a paired t-test. Frequency of MRSA in a hospital has been shown to be independently associated with rates of vancomycin use (19). We used linear regression modeling to determine which types of practice changes were independently associated with changes in vancomycin use in ICUs, after the data were adjusted for each ICU and changes in MRSA prevalence by the GLM procedure (SAS Institute Inc.). Detection of potential influential data points and their influence on main effect factors were also assessed in the modeling process. All reported p values are two-tailed. Analyses were repeated by using the relative change of each parameter rather than difference in rates.

## Results

### Description of Sites

During the study period, 21 hospitals representing 55 ICUs followed the surveillance protocol and reported at least 6 months of data by the time of the intervention and a median of 32 months (range 18–45) of data during the study period. Twenty (95%) hospitals completed the intervention survey representing the 50 ICUs included in this analysis. These hospitals were from 18 states and had a median hospital bed size of 351 (range 147–1,022); 13 (65%) reported an affiliation with a teaching institution (i.e., major teaching centers), and 2 (10%) were Veterans Affairs Medical Centers. The ICUs included 14 combined medical-surgical ICUs, 7 cardiac care units, 7 MICUs, 8 general SICUs, 6 cardiothoracic ICUs, 4 neurosurgical ICUs, 3 pediatric ICUs, and 1 burn ICU.

### Use of Local Data Compared with Benchmark

Infection-control teams at all hospitals disseminated the benchmark data to a variety of hospital committees and personnel (e.g., pharmacy and therapeutics, ICU personnel). This feedback was usually in the form of a committee report or memo. In addition to reporting, 12 (60%) hospitals changed prescribing of vancomycin (i.e., prescriber practice change). Eight (40%) hospitals reported at least one prescriber practice change (many hospitals combined several hospitalwide change in prescriber practice) that was applied hospitalwide; these changes encompassed 22 ICUs (Table). The hospitalwide changes included evaluating periodic drug use (19 ICUs), redistributing guidelines on appropriate uses of vancomycin (20 ICUs) by newsletter or mail (9 ICUs), and requiring prior approval for use of vancomycin (3 ICUs). In contrast, four hospitals reported focused (i.e., ICU-specific) practice changes in 11 ICUs; these included ICU-specific education in-service sessions on appropriate vancomycin use (8 ICUs) and removing vancomycin as routine prophylaxis for cardiac surgery (2 ICUs). Both practice changes were reported in one ICU (Table).

**Table:** Prescribing practice changes implemented in response to benchmark data intervention, and mean rate of vancomycin use^a^ before and after implementation, 50 Project ICARE ICUs, January 1996–July 1999^b^

Vancomycin use prescribing practice change	No. of ICUs (%)	Vancomycin use before and after practice change				p value^c^
	(n=50)	Change absent		Change present		
		Before	After	Before	After	
Hospitalwide^d^	22 (44%)					
Drug use evaluation	19 (38%)	74.2	80.5	105.3	94.1	0.62
Redistributed HICPAC guidelines on VRE	9 (18%)	79.4	84.6	116.0	90.6	0.34
Prior approval of vancomycin required	3 (6%)	87.2	99.4	84.7	67.2	0.25
Unit-specific^d^	11 (22%)					
ICU-specific education on appropriate vancomycin use	9 (18%)	75.9	96.3	83.3	132.1	0.01
Removed vancomycin from surgical prophylaxis	3 (6%)	82.0	82.2	85.9	149.1	0.01
^a^Defined daily doses per 1,000 patient-days. ^b^Abbreviations: ICARE, Intensive Care Antimicrobial Resistance Epidemiology; ICU, intensive-care units; HICPAC, Healthcare Infection control Practices Advisory Committee; VRE, vancomycin-resistant enterococci. ^c^Paired t-test. ^d^Components of each major categories are not mutually exclusive, so one ICU may be represented in several components of each category.						

### Vancomycin Use

**Figure 1:**
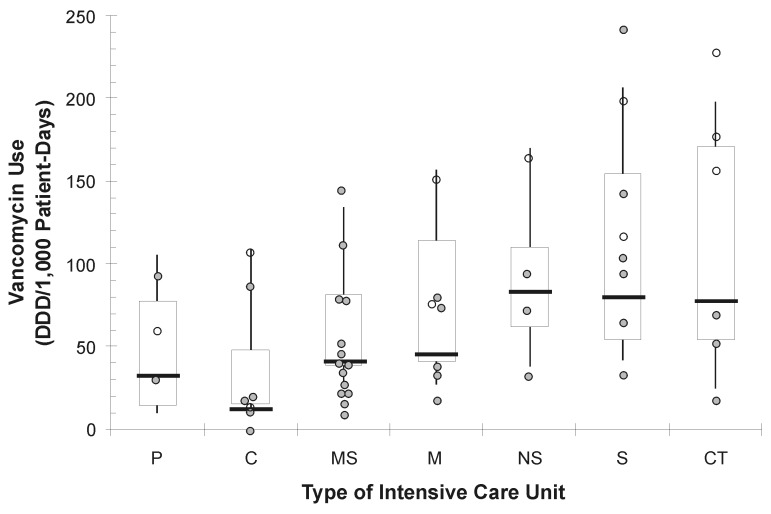
Boxplot of benchmark data of vancomycin use at all Phase 2 Project Intensive Care Antimicrobial Resistance Epidemiology (ICARE) hospitals (n=113 intensive-care units [ICUs]) in October 1997, by type of ICU (18). ICU types include pediatric (P), coronary (C), combined medical-surgical (MS), neurosurgical (NS), surgical (S), and cardiothoracic (CT). For each type of ICU, boxes represent rates of vancomycin use at the 25th–75th percentiles (interquartile range), and ends of vertical lines represent values at the 10th–90th percentiles. Horizontal lines represent median values in each ICU type. Additionally, plotted circles represent the rate of vancomycin use in the pre-intervention period (1996–1997) in the 50 ICUs participating in the intervention study, and open circles represent the 10 ICUs reporting a prescriber practice change identified in the specific unit (i.e., ICU-specific practice change) (1 burn ICU not shown). DDD, defined daily doses.

In the 50 study ICUs, the rates of vancomycin use during the pre-intervention period (Figure 1, plotted circles) were similar in range to the 113 ICARE Phase 2 ICUs contributing data to the national aggregate benchmark report (Figure 1, box plots). The overall (pooled mean) ICU-specific use of vancomycin in the 50 ICUs at the 20 study hospitals after the intervention was 89.1 defined daily doses per 1,000 patient-days, a 2.8% increase over the pre-intervention rate of use (86.6 defined daily doses per 1,000 patient-days). Despite this increase in aggregate usage among all ICUs, most ICUs reported lower rates of vancomycin use after the intervention compared with the pre-intervention rates. The median difference was -3 defined daily doses per 1,000 patient days (range -138 to +196), but this difference was not statistically significant.

Differences in the rate of vancomycin use varied substantially by the type of practice change. ICUs, in which unit-specific programs were implemented, used significantly lower rates of vancomycin in the postintervention period than in the pre-intervention period (Table), including both ICU-specific educational in-service (mean difference of -35.8 vs. +7.6, defined daily doses per 1,000 patient-days, p=0.01) and removal of vancomycin as routine surgical prophylaxis for cardiac surgery (mean difference -66.9 vs. +4.2 defined daily doses per 1,000 patient-days, p=0.01). In the multivariable analysis in which data were adjusted for each ICU and changes in MRSA prevalence, ICUs in which unit-specific practices were identified for improvement used, on average, 49 fewer daily doses of vancomycin per 1,000 patient days than did the other ICUs (parameter estimate -48.5; 95% confidence limits -68.8, -28.22; p=0.0001), compared to pre-intervention levels. The ICUs reported a 35%–37% decrease in median vancomycin use (median 132 daily doses of vancomycin per 1,000 patient days for unit-specific education and 149 for removal of prophylaxis) (Table). Analyses were repeated by using the relative change of each parameter rather than difference in rates, with similar results of statistical significance.

### VRE and MRSA

**Figure 2:**
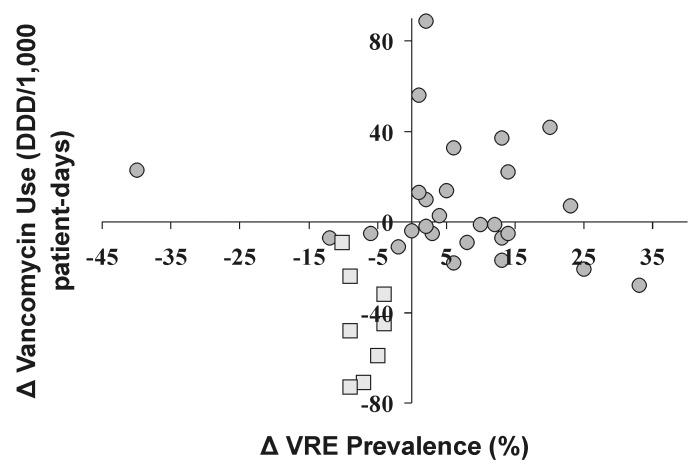
Difference (postintervention period minus pre-intervention) in rate of vancomycin use and prevalence of vancomycin-resistant enterococci (VRE) (%) in 35 intensive-care units (ICUs) testing >10 clinical isolates of *Enterococci* spp., Project Intensive Care Antimicrobial Resistance Epidemiology (ICARE), January 1996–July 1999. Squares represent ICUs reporting a prescriber practice change targeted in the specific ICUs (i.e., ICU-specific practice change). DDD, defined daily doses.

Thirty-five (70%) of the 50 study ICUs tested at least 10 isolates of enterococci for vancomycin susceptibility and were included in the calculations of VRE prevalence during both pre- and postintervention periods. During the pre-intervention period, these ICUs reported a median VRE prevalence of 11.7%. Overall, VRE prevalence increased during the postintervention period compared with the pre-intervention period among all study ICUs (median difference +2.3%; range –41% to +32%), although this difference was not statistically significant. However, when compared by type of practice change, the difference in VRE prevalence was significantly lower in ICUs in which unit-specific practice changes occurred, compared with other ICUs (mean difference -7.5% vs. +5.7%, p<0.001). Although many of the ICUs with decreases in vancomycin use reported increases in percent VRE, all the ICUs noting a unit-specific practice change reported decreases in both percent VRE and vancomycin use (Figure 2). Analysis of these data by using either the relative change in percent VRE or vancomycin use obtained results of similar statistical significance. However, since the relative changes were commonly of extreme values (range 0–400%), these are not reported here.

Because vancomycin use is associated with prevalence of MRSA in ICUs (19), we also evaluated temporal trends in MRSA prevalence. During the pre-intervention period, these ICUs reported a median MRSA prevalence of 33.5%. Overall, prevalence of MRSA increased during the postintervention period compared with the pre-intervention period in all study ICUs (mean difference +5.5%; range –22% to +38%; p=0.02). The increase in MRSA prevalence was similar in ICUs reporting unit-specific practice change compared with other ICUs (mean difference +2.7% vs. +7.1%, p=0.39).

## Discussion

In this study involving 50 ICUs from 20 hospitals, we evaluated the effect of inter-institution benchmarking of vancomycin use on reducing vancomycin use and prevalence of VRE. Our study suggests that hospital personnel can use local monitoring data, interpreted in the context of a risk-adjusted external benchmark, to help direct their efforts to reduce excessive use of antimicrobial drugs and reduce antimicrobial resistance. Having access to these data empowered the hospital personnel to make recommendations directed at the specific ICU. Our study further suggests that focused efforts (i.e, ICU specific) may be a more effective means to reduce excessive vancomycin use than hospitalwide activities.

The external benchmarks used were risk adjusted (i.e., stratified by ICU type) to account for the different rates of vancomycin used by different types of ICUs (18). Comparison of local data to a risk-adjusted benchmark should make the comparison more relevant (and more believable) to the ICU staff responsible for prescribing and other patient-care activities. Although several health-care reform proposals suggest some form of interfacility comparisons and public reporting of these types of data (21,22), caution must be exercised by ensuring that the comparisons are risk adjusted. We think part of the success of this study was that risk-adjusted comparisons were provided, rather than an overall single benchmark for all ICU types or all hospitals combined. These comparisons allowed hospital personnel to target unit-specific practice changes to particular ICU areas identified as having an excessive amount of vancomycin used compared with similar types of ICUs in the national benchmark. The reasons unit-specific change in prescriber practice in ICUs were associated with decreases in vancomycin use and VRE are not certain. The impact of the unit-specific practice changes may actually result from engaging local opinion leaders, as has been successfully done in other quality improvement projects (23,24).

Our study suggests that benchmarking rates of antimicrobial use, feedback on these rates, and changes in practice can lead to changes in antimicrobial use. However, the use of some overlapping practice changes and the absence of randomization may limit the ability to generalize the specific practice changes described in this study. In addition, hospitals began activities to reduce vancomycin use through changes in prescriber practice independent of the investigation. In fact, several of the ICUs that used unit-specific changes had the highest rates of vancomycin use, and this excessive use may have made the ICU staff more receptive to the change. These identified changes may not have been successful if implemented in ICUs in which the usage of vancomycin had not been as excessive. This theory may be true but does not detract from the major finding of this study: participation in a monitoring program with comparisons to a valid benchmark provided useful data and allowed the hospital to implement an effective change in practice. In addition, the retrospective nature of ascertaining the description of changes in prescriber practice may involve some recall bias. However, this study demonstrates how a monitoring system provides the tools for hospitals to make rational, valid decisions about initiating activities to change prescribing practices of vancomycin. One aspect of a quality improvement project that was missing from this study was the ability of the infection-control staff to share quality improvement protocols or ideas with other institutions participating in the monitoring system, as has been reported in other quality improvement studies using benchmarking (9).

Our study suggests that interpreting local data in the context of a risk-adjusted benchmark can aid in quality improvement decisions. Many of the study hospitals are continuing to voluntarily report data on antimicrobial use and antimicrobial resistance to CDC’s NNIS system as part of a continued quality improvement process. As hospital information systems become more automated, aggregating data such as these should become commonplace. Understanding how to best benchmark and respond to these data will be critical in our efforts to reduce antimicrobial-resistant infections.
